# Exploration of the SIRT1-mediated BDNF–TrkB signaling pathway in the mechanism of brain damage and learning and memory effects of fluorosis

**DOI:** 10.3389/fpubh.2023.1247294

**Published:** 2023-08-30

**Authors:** Feiqing Wang, Yanju Li, Dongxin Tang, Bo Yang, Tingting Tian, Mengxian Tian, Na Meng, Wei Xie, Chike Zhang, Zhixu He, Xiaodong Zhu, Dong Ming, Yang Liu

**Affiliations:** ^1^Academy of Medical Engineering and Translational Medicine, Tianjin University, Tianjin, China; ^2^Medical Research Center, The First Affiliated Hospital of Guizhou University of Traditional Chinese Medicine, Guiyang, Guizhou Province, China; ^3^Department of Hematology, Affiliated Hospital of Guizhou Medical University, Guiyang, Guizhou Province, China; ^4^National & Guizhou Joint Engineering Laboratory for Cell Engineering and Biomedicine Technique, Guiyang, Guizhou Province, China; ^5^Neurological Institute, Tianjin Medical University General Hospital, Tianjin, China

**Keywords:** fluorosis, brain damage, oxidative stress, inflammatory factors, SIRT1/BDNF/TrkB, learning and memory ability

## Abstract

**Introduction:**

Fluoride is considered an environmental pollutant that seriously affects organisms and ecosystems, and its harmfulness is a perpetual public health concern. The toxic effects of fluoride include organelle damage, oxidative stress, cell cycle destruction, inflammatory factor secretion, apoptosis induction, and synaptic nerve transmission destruction. To reveal the mechanism of fluorosis-induced brain damage, we analyzed the molecular mechanism and learning and memory function of the SIRT1-mediated BDNF–TrkB signaling pathway cascade reaction in fluorosis-induced brain damage through *in vivo* experiments.

**Methods:**

This study constructed rat models of drinking water fluorosis using 50 mg/L, 100 mg/L, and 150 mg/L fluoride, and observed the occurrence of dental fluorosis in the rats. Subsequently, we measured the fluoride content in rat blood, urine, and bones, and measured the rat learning and memory abilities. Furthermore, oxidative stress products, inflammatory factor levels, and acetylcholinesterase (AchE) and choline acetyltransferase (ChAT) activity were detected. The pathological structural changes to the rat bones and brain tissue were observed. The SIRT1, BDNF, TrkB, and apoptotic protein levels were determined using western blotting.

**Results:**

All rats in the fluoride exposure groups exhibited dental fluorosis; decreased learning and memory abilities; and higher urinary fluoride, bone fluoride, blood fluoride, oxidative stress product, and inflammatory factor levels compared to the control group. The fluoride-exposed rat brain tissue had abnormal AchE and ChAT activity, sparsely arranged hippocampal neurons, blurred cell boundaries, significantly fewer astrocytes, and swollen cells. Furthermore, the nucleoli were absent from the fluoride-exposed rat brain tissue, which also contained folded neuron membranes, deformed mitochondria, absent cristae, vacuole formation, and pyknotic and hyperchromatic chromatin. The fluoride exposure groups had lower SIRT1, BDNF, and TrkB protein levels and higher apoptotic protein levels than the control group, which were closely related to the fluoride dose. The findings demonstrated that excessive fluoride caused brain damage and affected learning and memory abilities.

**Discussion:**

Currently, there is no effective treatment method for the tissue damage caused by fluorosis. Therefore, the effective method for preventing and treating fluorosis damage is to control fluoride intake.

## 1. Introduction

Fluorosis is a systemic disease caused by long-term intake of excessive amounts of fluoride. Fluorosis occurs in more than 50 countries and regions worldwide, which include China, India, Bangladesh, Algeria, Thailand, Iran, Argentina, the United States, Canada, Vietnam, Mexico, Sri Lanka, Morocco, Egypt, and South Africa (1). Fluorosis is a global public health problem, as >260 million people worldwide obtain drinking water from sources with high fluoride concentrations (2). China is severely affected by fluorosis, where varying degrees of fluorosis occur in almost all provinces and autonomous regions, and the population under threat numbers 110 million (3, 4). Consequently, fluorosis is currently an important health issue in China.

Fluorosis not only significantly damages bone and teeth, but also causes varying degrees of damage to non-bone tissues such as the liver, kidneys, gastrointestinal tract, nervous system, cardiovascular system, endocrine system, and reproductive system (5–7). Due to the lack of effective treatment drugs for fluorosis, people in fluorosis areas remain vulnerable to fluorosis. Therefore, medical research is currently focused on fluorosis pathogenesis and prevention measures.

Fluorine is an element with extremely active chemical properties. Excessive fluoride intake might directly attack oxygen, interfere with oxygen metabolism, and lead to increased oxygen free radicals (8, 9). Simultaneously, fluoride can also decrease antioxidant enzyme activity and non-enzymatic antioxidant substance content, all of which lead to excessive oxygen free radical generation (10, 11). Fluoride free radicals can attack the covalent bond of unsaturated fat acids, which causes lipid peroxidation and increases the free radicals in the body (12). The free radical metabolism imbalance caused by fluorosis and the accumulation of numerous oxidative stress products leading to organ dysfunction are a perpetual hot research topic. Oxidative stress damage also promotes inflammatory factor production in the body (13, 14). Currently, oxidative stress inflammatory factor levels have been identified as biological markers of fluorosis damage in the body (15).

Fluoride can enter the brain through the blood–brain barrier, and long-term intake of excessive fluoride can lead to fluoride accumulation in the brain, which affects the normal physiological function of brain cells (16). Fluoride accumulation can damage the nervous system, which includes neuropathological changes, cholinergic nervous system abnormalities, nerve cell membrane structure changes, neurotransmitter receptor changes, neurocyte apoptosis, and decreased intelligence (17). The change in the intelligence of children in endemic fluorosis areas was confirmed in several countries (18). Excess fluoride can elicit damaging effects in humans of any age, where the toxic damage is irreversible, especially neurotoxicity in developing children (19).

**Figure 1:**
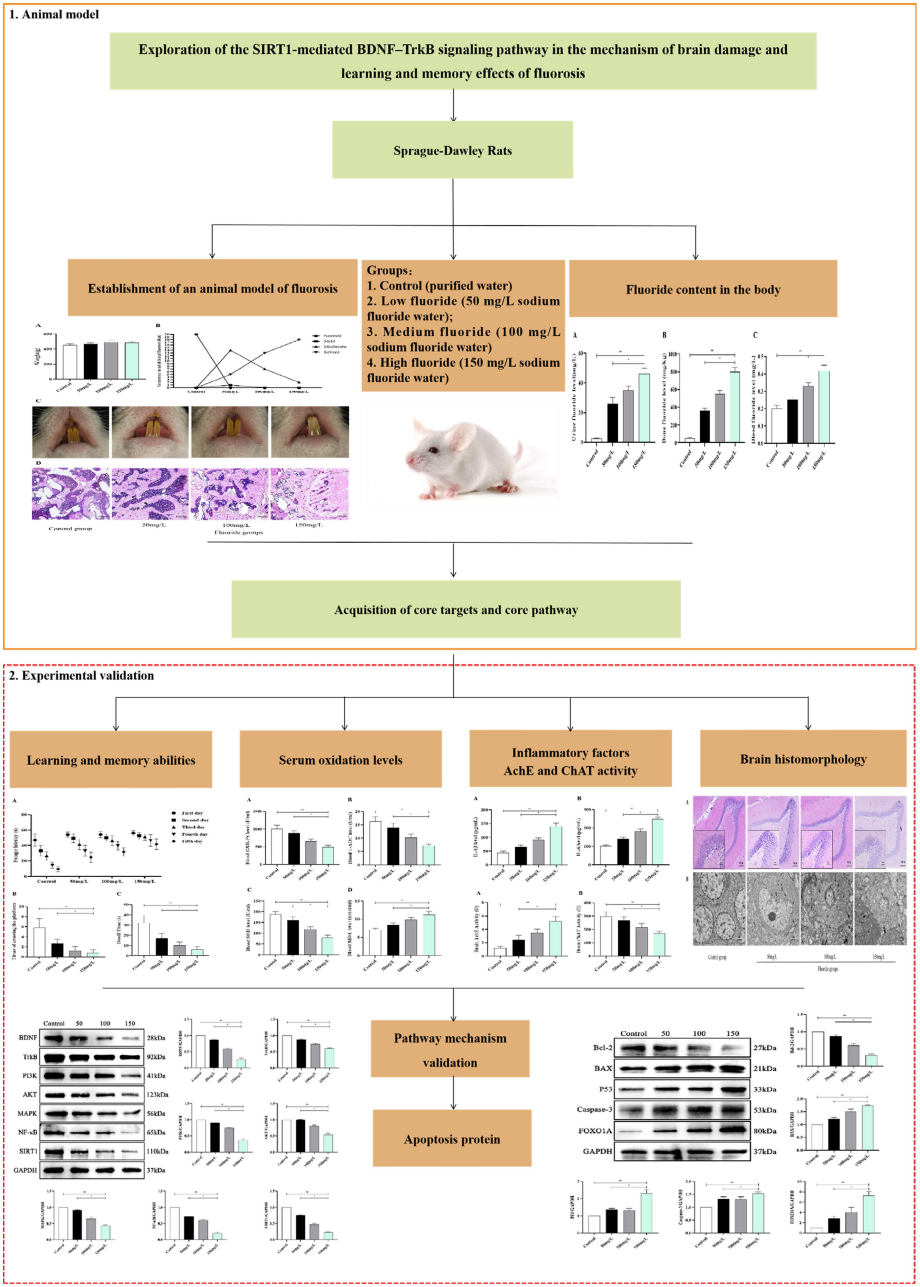
Flowchart of brain injury in fluorosis.

Fluoride is also an important substance in the signal transduction pathway (20). Brain cell apoptosis can be induced by reducing the expression of silent information regulator 1 (SIRT1) and brain-derived neurotrophic factor (BDNF), where SIRT1 regulates the downstream BDNF (21). SIRT1 participates in oxidative stress, the inflammatory response, neuroprotection, and other effects through deacetylation (22). BDNF is mainly distributed in the central nervous system and participates in neural survival and protection, where elevated BDNF levels improve learning and memory dysfunction (23). Upon binding with receptor tyrosine protein kinase B (TrkB) as a ligand, BDNF activates the intracellular TrkB and its downstream signaling protein phosphatidylinositol 3-kinase–protein kinase B (PI3K–Akt) pathway (24). The PI3K–Akt pathway activation activates mitogen-activated protein kinase–nuclear factor kappa B (MAPK–NF-κB) multiple cascade reactions. These pathways control nerve cell survival, growth, and differentiation (25). BDNF–TrkB protect brain histiocytes from damage and participate in learning and memory formation. Currently, there is little research on the mechanisms related to fluorosis and brain injury. Therefore, we analyzed the relationship between the SIRT1-mediated BDNF–TrkB signaling pathway and fluorosis brain injury, learning and memory, and provide new ideas and a theoretical basis for preventing and treating fluorosis brain injury. A flowchart of this study is shown as Figure 1.

## 2. Methods and materials

### 2.1. Construction of the animal model

We used specific pathogen-free male Sprague–Dawley rats (100–120 g, two weeks after weaning) provided by the Animal Experiment Center of Guizhou Medical University [certificate number SCXK (Qian) 2021–0001], Guiyang, China. The rats were housed in a room with 20–25°C ambient temperature and 60% ± 20% humidity, and exposed to a 12-h light–dark cycle.

The fluoride exposure dose design referred to sub-chronic toxicity experiment requirements, where the highest dose was typically 5–20% of the median lethal dose (LD50) of the test substance. The LD50 concentration of fluoride was 1,500 mg/L. Based on previous experimental results and the literature, the sodium fluoride exposure doses in this study were 50 mg/L, 100 mg/L, and 150 mg/L (Zhanwang Chemical Reagents, Wuxi, China). The rats were fed adaptively for 1 week and divided randomly into four groups (80 rats in total, 20 in each group): control (purified water), low fluoride (50 mg/L sodium fluoride water), medium fluoride (100 mg/L sodium fluoride water), and high fluoride (150 mg/L sodium fluoride water). The rat growth and development were documented over 90 days of fluoride exposure. Then, the rats were killed after anesthesia induction, and the blood and brain tissue were harvested rapidly and stored at −80°C. All animal experiments were approved by the First Affiliated Hospital of Guizhou University of Traditional Chinese Animal Ethics Committee (approval number: AHQU20210515A).

### 2.2. Test indicators

#### 2.2.1. Body weight

Before the rats were killed, they were weighed using an electronic balance (Huazhi Scientific Instruments, Beijing, China) for 10 consecutive days and their body weights were recorded.

#### 2.2.2. Dental fluorosis

Changes in the rat teeth were observed every week to determine the degree of damage, where the teeth were photographed before they were killed. According to the Diagnostic Standard for Dental Fluorosis (WS-T208-2011) in China, dental fluorosis is classified into four grades: normal (translucent and milky white enamel, and smooth and shiny surface), mild (small, chalky opaque areas on the tooth surface, some parts of the tooth surface are worn, and the maxillary anterior teeth are occasionally blurred and colored), moderate (a chalky opaque area covers the entire tooth surface, independent honeycomb-like defects can be seen, and teeth have obvious wear), and severe [enamel surface is seriously affected, obviously underdeveloped, enamel defect is fused (in bands or sheets), tooth surface is widely colored, the color can vary from brown to nearly black, and the tooth often presents an erosion-like appearance].

#### 2.2.3. Learning and memory ability evaluation

The Morris water maze (MWM) experiment was conducted 7 days before the end of the experiment, and was divided into the directional navigation experiment and space exploration experiment. The water tank was 120 cm in diameter and 50 cm high, the water was 30 cm deep, and the water temperature was maintained at 26°C ± 1°C. Four equidistant points [north (N), east (E), south (S), west (W)] were marked on the pool wall as the experiment starting points. The pool was divided into four quadrants (NW, WS, SE, EN), with any one quadrant placed in the center of the platform (the platform was equidistant from the center of the pool wall). The platform was colorless and transparent (diameter: 12 cm, height: 29 cm), and was submerged 1 cm underwater. Two bags of milk powder were dissolved in each experimental water to render the platform invisible. Rich reference clues were pasted around the pool (such as triangles, squares, circles, diamonds, and other geometric shapes in various quadrants) and remained unchanged for the rats to use to locate the platform. Silence and consistent lighting were maintained during the experiment.

For the navigation experiment, the rats were placed in the water from different quadrants facing the pool wall, and the time (escape latency) and navigation trajectory required for rats to find the platform from their entry point were observed. If the rat failed to find the platform within 60 s, it was artificially led to the platform and remained there for 15 s. At this time, the incubation period was calculated as 60 s, and the computer intelligently recorded the results for five consecutive detection days (MWM Image Automatic Acquisition and Processing System, Institute of Pharmacy, Chinese Academy of Sciences, Beijing, China).

On day 6 of the space exploration experiment (to test the rats’ memory regarding the original platform), the platform was removed, and the rats were placed in the water from the opposite quadrant of the platform. The number of times the rats crossed the platform within 60 s and the duration they remained in the quadrant where the platform was located were recorded.

#### 2.2.4. Skeletal pathology

The right femoral head was harvested after the rats had been killed. The muscle tissue was removed carefully, then the femur was washed with phosphate-buffered saline and fixed in 4% paraformaldehyde solution for 24 h. Subsequently, the femur was removed from the paraformaldehyde solution, washed thrice with distilled water, transferred to EDTA-containing decalcification solution, and treated for 3–4 weeks. The bone tissue was removed from the decalcification solution after it had softened or there was no resistance to a needle. Then, the bone tissue was washed with distilled water for 3–4 min, dehydrated, embedded in paraffin, sectioned, and stained (hematoxylin–eosin, HE). Morphologic changes in the bone tissue were observed under a light microscope (Leica, Wetzlar, Germany) at ×200 magnification.

#### 2.2.5. Urinary fluoride level

Before the rats were killed, the urine excreted over 24 h was collected while rats were in a metabolic cage. Then, 10 mL urine was placed in a urine sediment tube and centrifuged (1,500 × *g*, 5 min, room temperature). The supernatant was collected, and the fluoride content was measured using a fluoride ion-selective electrode (Precision Scientific Instruments, Shanghai, China).

#### 2.2.6. Bone fluoride level

The right femur was removed after the rats had been killed, and the muscles and fat were removed. Then, the femur was dried in a 105°C oven. After high-temperature ashing, the fluoride level in the femur was measured using a fluoride ion-selective electrode (Precision Scientific Instruments).

#### 2.2.7. Serum fluoride level

Blood was collected from the rat femoral artery following anesthesia induction. The sample was placed on ice for 30 min, centrifuged (1,500 × *g*, 10 min, room temperature), and the serum was stored at low temperature. The fluoride level in the blood was determined using a fluoride ion-selective electrode (Precision Scientific Instruments).

#### 2.2.8. Oxidation product level in blood

Blood was collected from the rat femoral artery after anesthesia induction, placed on ice for 30 min, centrifuged (1,500 × *g*, 10 min, room temperature), and the serum was stored at low temperature. The level of glutathione peroxidase (GSH-Px) was determined by the 5,5′-dithiobis (2-nitrobenzoic acid) method. Total antioxidant capacity (T-AOC) was determined by the Fe^3+^ reduction method. Superoxide dismutase (SOD) activity was measured by the xanthine oxidase method. Malonaldehyde (MDA) content was determined by the thiobarbituric acid method. The assay kits were from Jiancheng Bioengineering Institute (Nanjing, China) and the specific experimental steps were conducted in strict accordance with the manufacturer’s instructions.

#### 2.2.9. Inflammatory factor levels in the brain

After the rats had been killed, the brain tissue was quickly removed. Physiological saline solution was added in a mass-to-volume ratio of 1:9 and whole brain tissue homogenized on ice cubes. The mixture was thoroughly mixed and centrifuged (1,500 × *g*, 10 min, room temperature), and the supernatant was aspirated. The interleukin-1β (IL-1β) and IL-6 levels were measured using a double antibody one-step sandwich enzyme-linked immunosorbent assay (ELISA). The assay kit was from Jiancheng Bioengineering Institute, and the specific experimental steps were carried out in strict accordance with the manufacturer’s instructions.

#### 2.2.10. Acetylcholinesterase (AchE) and choline acetyltransferase (ChAT) activity in the brain

After the rats had been killed, the brain tissue was quickly removed. Physiological saline solution was added in a mass-to-volume ratio of 1:9 and hippocampal tissue homogenized on ice cubes. After centrifugation, 0.8 mL supernatant was removed, 1.4 mL purified water was added, and mixed well. Then, 0.2 mL physostigmine sulfate (1.54 mmol/L) was added, and 0.8 mL trichloroacetic acid (1.84 mol/L) was slowly added in drops. The mixture was thoroughly mixed and centrifuged (1,500 × *g*, 10 min, room temperature), and the absorbance of the supernatant was determined using a UV–visible spectrophotometer with 540 nm wavelength and 1-cm diameter, where purified water was used as the zero. AchE and ChAT in the brain tissue were detected using the colorimetric method in strict accordance with the requirements of the reagent manual for specific steps. The assay kits were from Jiancheng Bioengineering Institute.

#### 2.2.11. Pathologic morphology of the brain

After the rats had been killed, the brain tissue was quickly removed and fixed in polyformaldehyde solution for 2 days. Subsequently, the tissue was dehydrated, embedded in paraffin, sectioned, and stained with HE. The morphologic changes in the brain tissue were observed under a light microscope (Leica) at ×200 magnification.

#### 2.2.12. Brain ultrastructure

After the rats had been killed, the brain tissue containing the hippocampus was quickly removed and placed on ice. Then, ~1 mm brain tissue was excised with a sharp blade and immersed immediately in fixation solution (3% glutaraldehyde, 1.5% polyformaldehyde, 0.1 M phosphate-buffered saline, pH 7.2) at 4°C for several days or ≥ 2 h. Ultrathin (70–80 nm) sections were prepared after immersion and embedding with resin (Epon 812; Solarbio, Beijing, China). The sections were stained with uranium dioxide acetate and lead citrate, and photographed under a transmission electron microscope (H-600; Hitachi, Tokyo, Japan).

#### 2.2.13. Western blotting

After the rats had been killed, the brain tissue containing the hippocampus was quickly removed. Total protein was extracted from the hippocampus using protease inhibitors, phosphatase inhibitors, phenylmethylsulfonyl fluoride (Solarbio), and radioimmunoprecipitation assay lysis buffer. Equal amounts of protein were separated by sodium dodecyl sulfate–polyacrylamide gel electrophoresis using a Solarbio system on 6–12% gels. Then, the proteins were transferred onto polyvinylidene fluoride (PVDF) membranes (0.45 μM; Millipore, Bedford, MA, USA), which were blocked with 5% skimmed milk for 1 h at room temperature. The PVDF membranes were then probed with primary antibodies (1:1000 dilution, all from Beyotime Biotechnology, Shanghai, China) against the following proteins: BDNF, TrkB, SIRT1, BAX, B-cell lymphoma (Bcl)-2, caspase-3, forkhead box protein O1 (FOXO1A), glyceraldehyde-3-phosphate dehydrogenase (GAPDH), NF-κB, PI3K, Akt, and MAPK. Then, the membranes were probed with goat anti-rabbit and goat anti-mouse horseradish peroxidase-coupled secondary antibodies (1,5,000 dilution; Boster Biotechnology, Wuhan, China) and incubated overnight at 4°C. The next day, the PVDF membranes were rinsed thrice with Tris-buffered saline–Tween 20 (Solarbio) and incubated with goat anti-rabbit or goat anti-mouse horseradish peroxidase-conjugated secondary antibodies for 2 h at room temperature. Finally, an ultra-sensitive electrochemiluminescence reagent was used with substrates (Boster Biotechnology) on the immune-responsive protein bands. The protein bands were quantified using ImageJ (National Institutes of Health, Bethesda, MD, USA) according to gray values normalized to the GAPDH level.

### 2.3. Statistical analyses

Statistical evaluations were conducted using Prism 8.3.1 (GraphPad, San Diego, CA, USA). Data are the mean ± SD. One-way ANOVA was used for inter-group comparisons, and further multiple comparisons were conducted. LSD-t test was used for those with uniform variance, and Dunnett T3 analysis was used for those with uneven variance. *p* < 0.05 was considered significant.

## 3. Results

### 3.1. Establishment of a rat model of fluorosis

**Figure 2:**
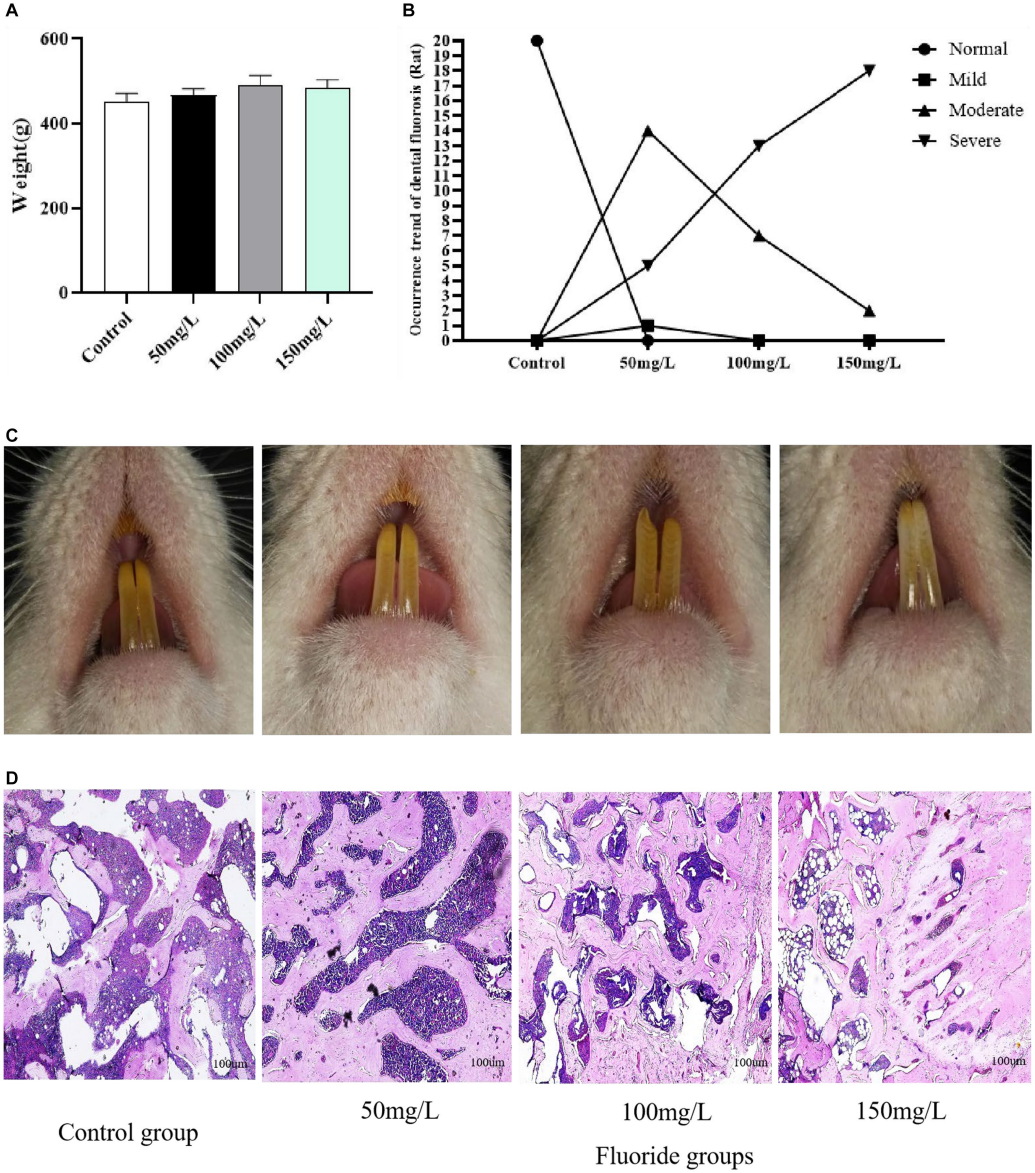
Establishment of an animal model of fluorosis. **(A)** Rat body weights (n = 10). **(B)** Number of rats with dental fluorosis (*n* = 20). **(C)** Degree of dental fluorosis in the rats. **(D)** HE-stained pathologic section of rat femur (Scale bar = 100 μm, ×40, *n* = 10).

The fluoride-exposed rats had greater body weights than the control rats, but these differences were not significant (*p* > 0.05) (Figure 2A). The control rats did not have dental fluorosis, while all fluoride-exposed rats did. The dental fluorosis was mainly moderate to severe as the fluoride exposure dose increased. The proportion of light, medium, and severe dental fluorosis in the fluoride-exposed groups (50 mg/L, 100 mg/L, 150 mg/L) was 5, 70, 25% (*n* = 1, 14, 5), 0, 35, 65% (*n* = 0, 7, 13), and 0, 10, 90% (*n* = 0, 2, 18), respectively (Figures 2B,C). Light microscopy revealed that the control rat femoral trabecular bone was arranged in an orderly manner, well connected, moderate in number, and had uniform thickness. Compared with the controls, the femoral trabecular bone of the fluoride-exposed rats was disordered, thickened, with smaller spacing. Furthermore, the bone damage gradually became severe as the fluoride dose increased (Figure 2D).

### 3.2. Fluoride level

**Figure 3:**
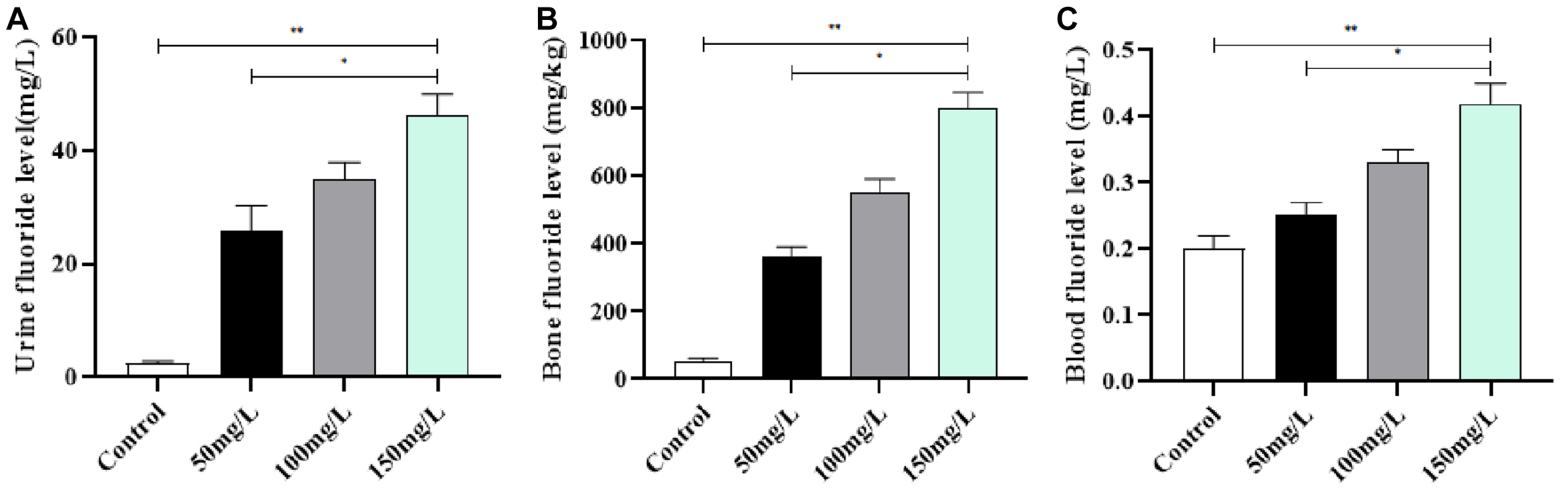
Fluoride content in the body. **(A)** Urinary fluoride level. **(B)** Fluoride level in bone. **(C)** Fluoride level in Serum. **p < 0.05 vs. control group, *p < 0.05 vs. fluoride group, *n* = 10.

Compared with the control group, the fluoride-exposed groups had significantly higher fluoride levels in the urine, bone, and serum (*p* < 0.05). Furthermore, the fluoride levels in the urine, bone, and blood of the exposed rats gradually increased as the fluoride intake increased, and the differences were statistically significant (*p* < 0.05) (Figure 3).

### 3.3. Learning and memory abilities

**Figure 4:**
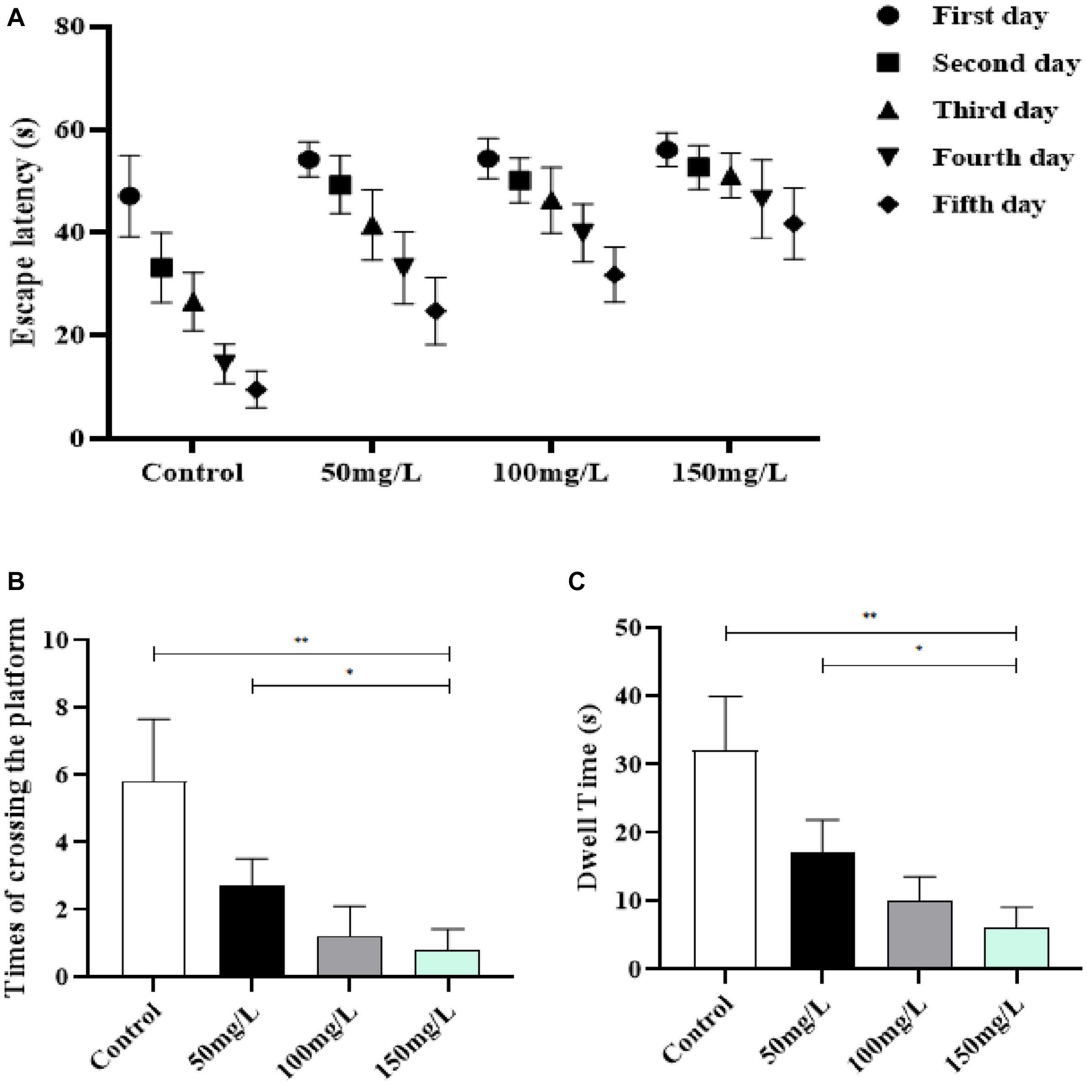
Effect of fluorosis on rat learning and memory abilities. **(A)** Escape latency. **(B)** Times platform was crossed. **(C)** Dwell time. **p < 0.05 vs. control group, *p < 0.05 vs. fluoride group, *n* = 10.

The learning and memory abilities of brain-injured rats were examined with the MWM experiment. The positioning navigation test demonstrated that the fluoride-exposed rats had significantly prolonged escape latency as compared with the control rats (*p* < 0.05) (Figure 4A). The space exploration experiment demonstrated that the fluoride-exposed rats required a significantly longer time to cross the platform for the first time, crossed the platform significantly fewer times, and required a significantly shorter time to cross the platform area as compared with the control rats (*p* < 0.05) (Figures 4B,C). Among the fluoride-exposed rats, the initial platform crossing was gradually prolonged, and the number of times the platform was crossed and the quadrant residence time at the original platform area gradually decreased as the fluoride exposure dose increased. The differences between the medium-, high-, and low-fluoride groups were statistically significant (*p* < 0.05), which demonstrated a dose–effect relationship (Figure 4).

### 3.4. Oxidation factors in the serum

**Figure 5:**
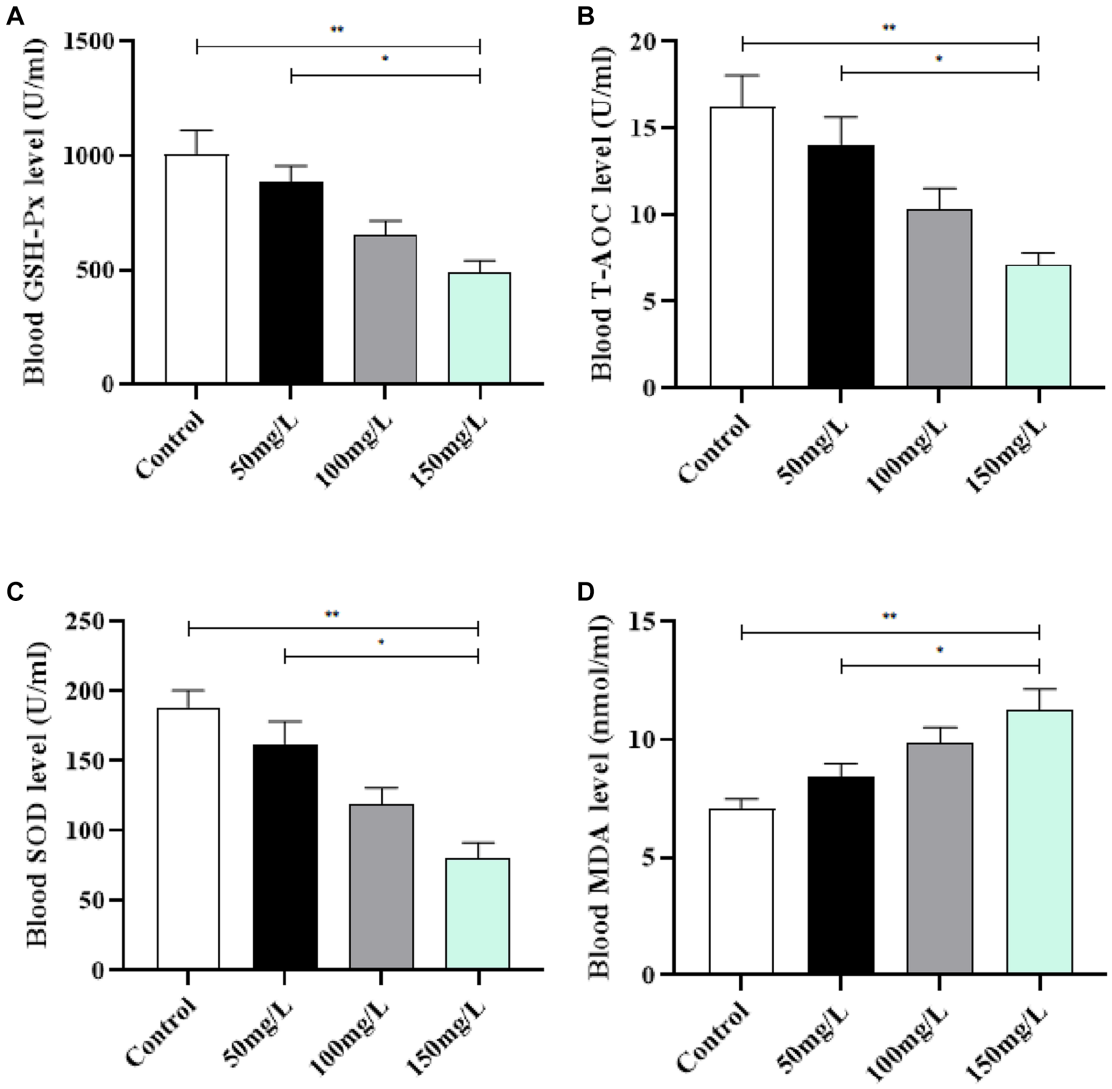
Effect of fluorosis on rat serum oxidation levels. **(A)** GSH-Px level. **(B)** T-AOC level. **(C)** SOD level. **(D)** MDA level. **p < 0.05 vs. control group, *p < 0.05 vs. fluoride group, *n* = 10.

Compared with the control group, the serum in the fluoride-exposed groups had significantly decreased GSH-Px, T-AOC, and SOD activity (*p* < 0.05) (Figures 5A–C). The fluoride-exposed rats had significantly higher serum MDA content than the control rats (*p* < 0.05) (Figure 5D). Among the fluoride-exposed groups, the serum GSH-Px, T-AOC, and SOD levels gradually decreased as the fluoride dose increased, while the MDA levels gradually increased, with statistically significant differences (Figure 5).

### 3.5. Inflammatory factor levels in brain tissue

**Figure 6:**
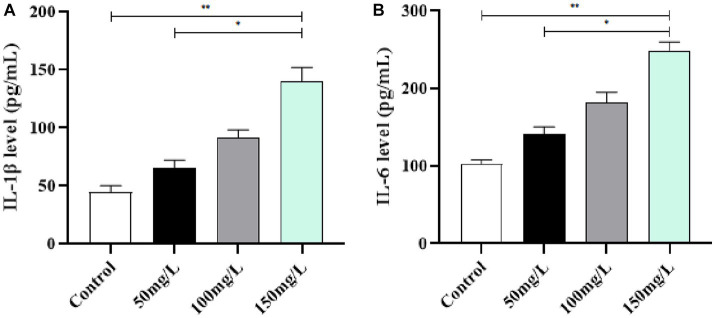
Effect of fluorosis on inflammatory factors in rat brain tissue. **(A)** IL-1β level. **(B)** IL-6 level. **p < 0.05 vs. control group, *p < 0.05 vs. fluoride group, *n* = 10.

Compared with the control rats, the brain tissue of fluoride-exposed rats had significantly increased IL-1β and IL-6 levels (*p* < 0.05) (Figures 6A,B). Among the fluoride-exposed groups, IL-1β and IL-6 gradually increased as the fluoride exposure dose increased, with statistical significance (*p* < 0.05). The results demonstrated that the gradual increase of IL-1β and IL-6 positively correlated with the fluoride exposure dose (Figure 6).

### 3.6. AchE and ChAT activity in brain tissue

**Figure 7:**
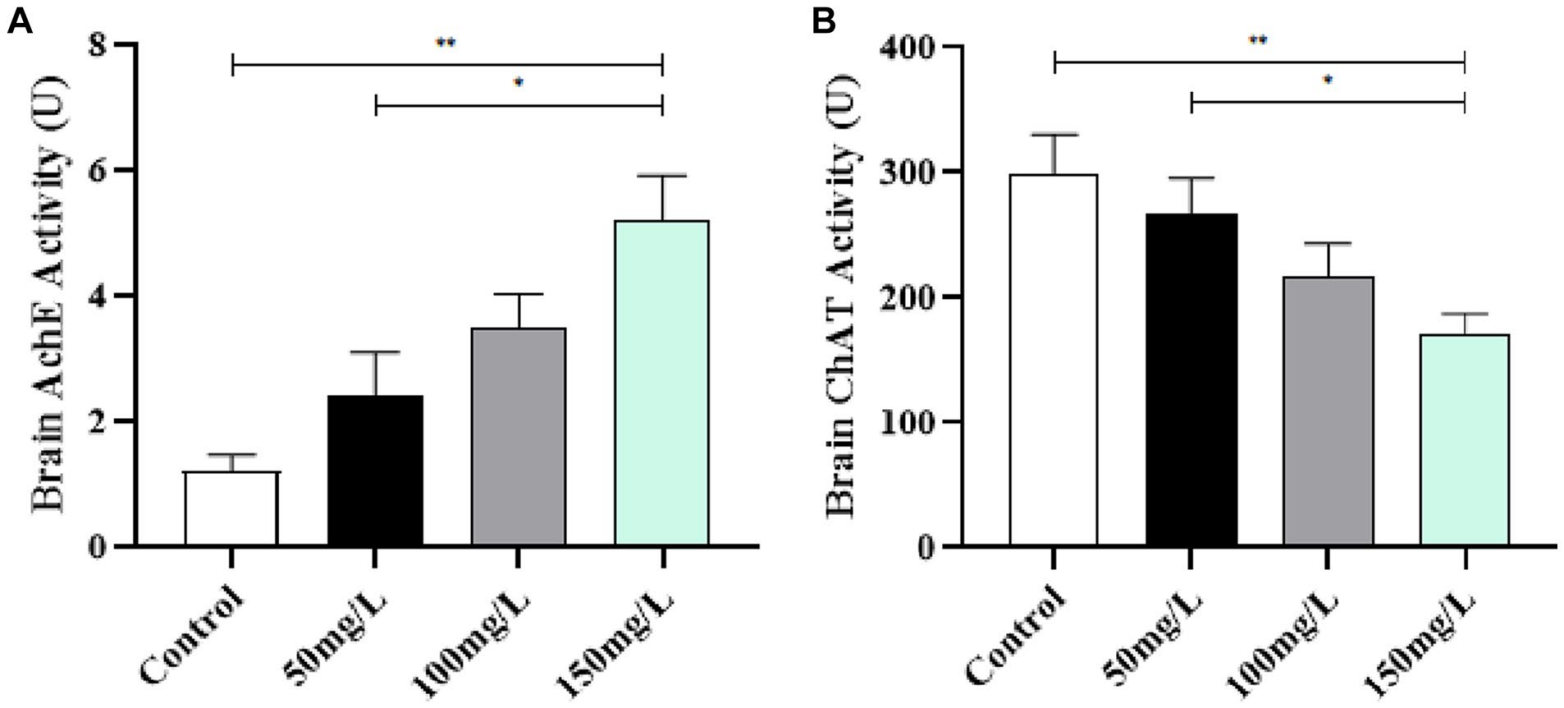
Effect of fluorosis on AchE and ChAT activity in rat brain tissue. **(A)** AchE level. **(B)** ChAT level. **p < 0.05 vs. control group, *p < 0.05 vs. fluoride group, *n* = 10.

Compared with the control group, the fluoride-exposed rat brain tissue had significantly increased AchE activity and significantly reduced ChAT activity (*p* < 0.05). Among the fluoride-exposed groups, the brain tissue AchE and ChAT activity was closely related to the fluoride exposure dose (Figure 7).

### 3.7. Brain tissue structure

**Figure 8:**
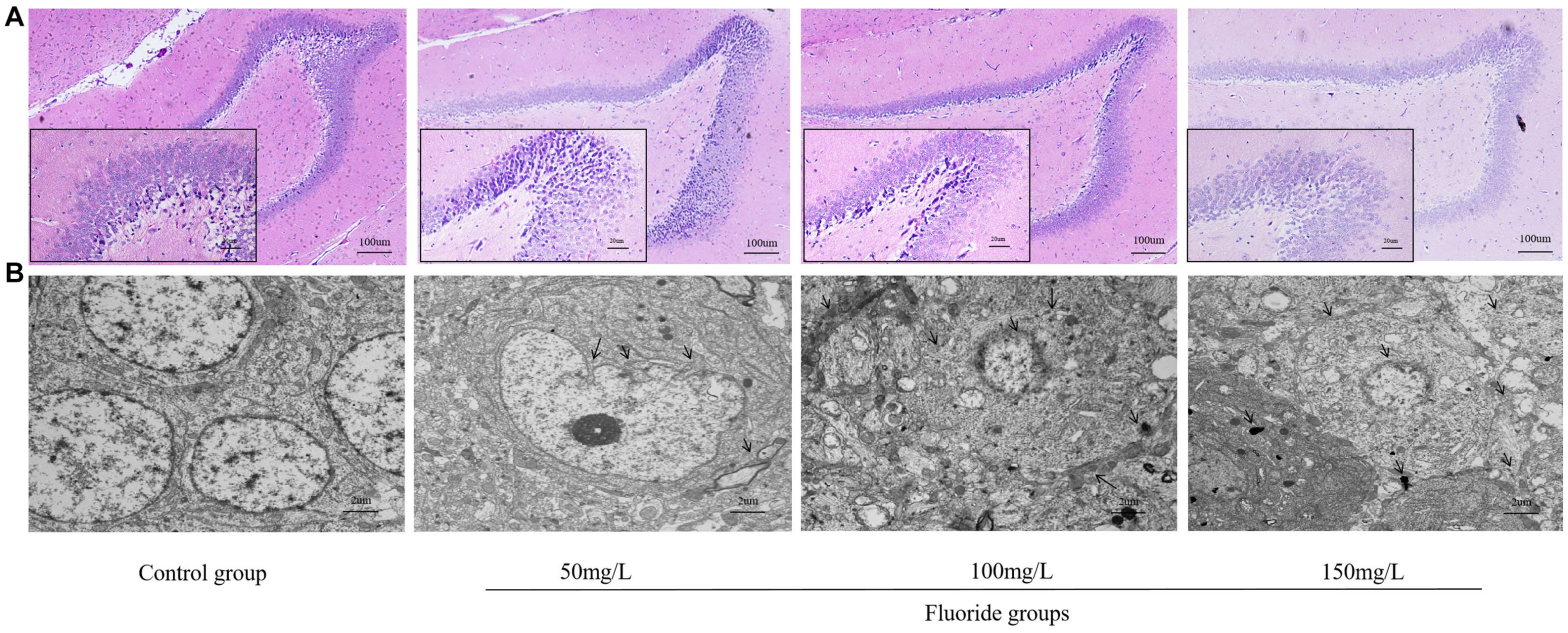
Effects of fluorosis on rat brain tissue structure. **(A)** HE staining of brain tissue exhibiting pathologic morphology (Scale bar = 20 μm, ×200, Scale bar = 100 μm, ×40, *n* = 10). **(B)** Brain tissue ultrastructure (Scale bar = 2 μm, ×6,000, *n* = 10).

The HE staining revealed that the control rat hippocampal neurons were arranged in a dense and ordered manner, with regular morphology, clear cell boundaries, rich cytoplasm, regular astrocytes, and clear nucleoli. Contrastingly, the hippocampal neurons of the fluoride-exposed rats were arranged sparsely, the cell boundary was not clear, the number of astrocytes was reduced significantly, cells were swollen, and the nucleoli had disappeared (Figure 8A). Transmission electron microscopy revealed that the control group had regularly shaped neuron nuclei, clear double-layer structure of the cellular nuclear membrane, evenly distributed nuclear chromatin, and normal distribution of several organelles. However, the fluoride-treated groups had folded neuron membranes, deformed mitochondria, absent cristae, vacuole formation, and pyknotic and hyperchromatic chromatin (Figure 8B).

### 3.8. Neurotrophin signaling pathway expression

**Figure 9:**
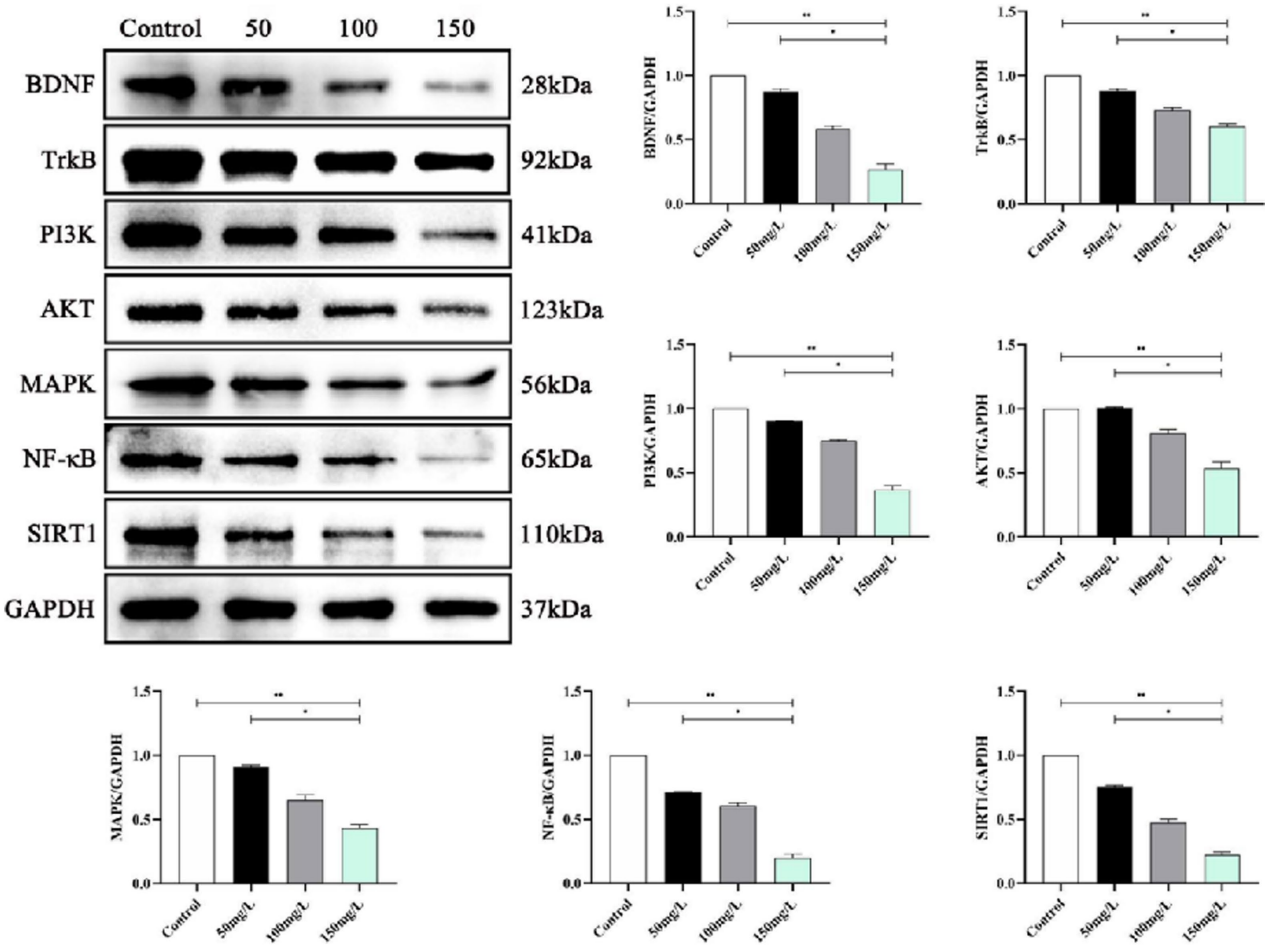
SIRT1 mediates protein expression of the BDNF–TrkB signaling pathway (*n* = 10).

**Figure 10:**
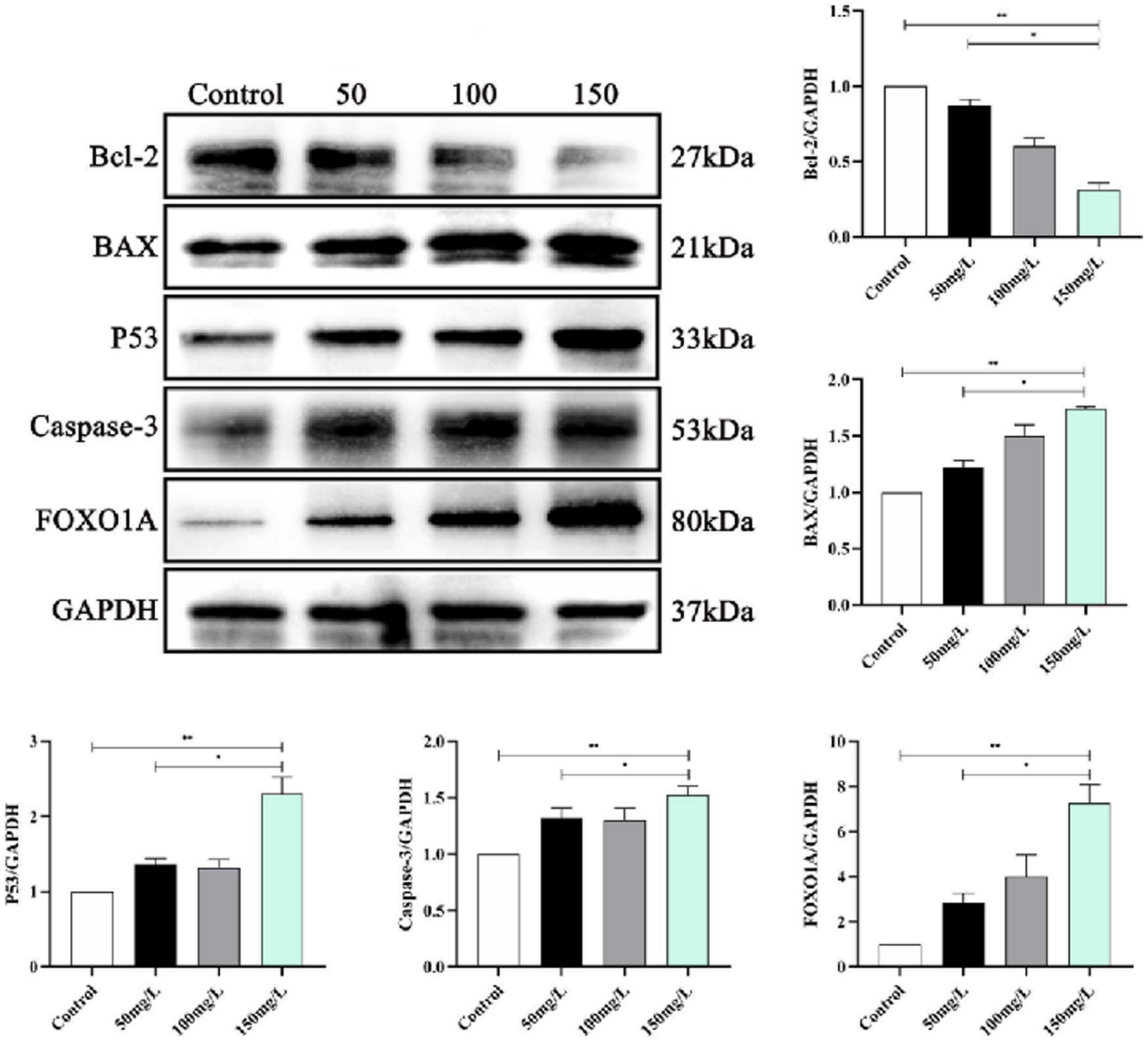
Expression of apoptotic proteins in rat brain injury caused by fluorosis exposure (*n* = 10).

We analyzed the SIRT1-mediated BDNF–TrkB pathway in the fluorosis rat brain tissue. Western blotting demonstrated that the fluorosis rat brain tissue had significantly decreased SIRT1, BDNF, TrkB, PI3K, Akt, MAPK, and NF-κB, protein expression (Figure 9) and significantly increased expression of apoptotic proteins (Bcl-2, BAX, Caspase-3, P53, FOXO1A) (Figure 10). The results indicated that SIRT1 mediated the BDNF–TrkB signaling pathway and participated in the mechanism of brain damage caused by fluorosis, and was closely related to the fluoride exposure dose.

## 4. Discussion

The mechanism of multiple organ and system damage caused by fluorosis is very complex and has always been a key public health issue of research concern. There are various sources of fluoride exposure in nature, such as food, water, air, and toothpaste, among which drinking water is the most important source (26). The fluoride from food and drinking water is absorbed by the gastrointestinal tract and transported to various tissues of the body through the systemic circulation. Most of ingested fluoride is deposited in calcified tissues such as the teeth and bones, and the remainder is distributed in vascular-rich soft tissues and blood (27). The kidneys are the main fluoride excretion pathway, and can eliminate 50–70% of fluoride *via* urine excretion (28). Therefore, dental fluorosis and urinary and bone fluoride concentrations are important indicators of fluorosis. Our results demonstrated no dental fluorosis in the control group, mild to moderate dental fluorosis in the low-fluoride group, and severe dental fluorosis the medium– and high-fluoride groups. Furthermore, the fluoride-exposed groups had significantly higher urinary and bone fluoride levels than the control group. The urinary and bone fluoride levels increased as the fluoride dose increased. The results indicated that the degree of dental fluorosis damage and urine and bone fluoride content are closely related to the fluoride exposure dose. Moreover, the HE staining of the bone tissue proved that the degree of bone damage was also related to the fluoride exposure dose. Therefore, the most effective means of preventing and treating fluorosis in the future is to reduce fluoride intake.

The MWM was invented by British psychologist Morris in 1981 and is used to study learning and memory in animals (29). The MWM has become a standard model for studying spatial learning and memory, and has been used in various studies, specifically for measuring rodent spatial memory and working memory (30). In 1937, first reported the manifestation of nervous system dysfunction in patients with endemic fluorosis. Since then, other studies have found that long-term excessive intake of fluoride can cause demyelinating changes in the cerebral cortex and subcortical areas and lead to hypothyroidism; this may explain the decline in the intelligence level of children in high fluoride areas. In this study, we constructed animal models of fluorosis, where the rats’ learning and memory abilities decreased as the fluoride dose increased, and were closely related to the fluoride dose. Long-term intake of excessive fluoride can directly affect the learning and memory abilities of the brain, which seriously affects the health of people in fluorosis areas. Most studies have reported that the IQ of children with long-term high fluoride intake was significantly lower than that of children with normal fluoride intake (31).

Protective mechanisms against reactive oxygen species toxicity form within cells during biological systems evolution. Among them, SOD widely exists in organisms, is important, and has obvious antioxidant effects (32). The oxidative stress theory systematically explains the pathogenesis of systemic damage caused by fluorosis, which many scholars at home and abroad have acknowledged and is a current hot research topic in medicine (33). Brain tissue is highly dependent on oxygen and rich in polyunsaturated fatty acids that are easily attacked by free radicals, which cause lipid peroxidation and oxidative stress damage (34, 35). Fluorosis increased oxidative stress levels, which led to apoptosis, and the decreased learning and memory abilities of fluorosis rats were related to the increased oxidative stress levels (36, 37). Therefore, the brain is the organ that receives the most obvious damage from free radicals during fluorosis. In this study, the fluorosis rats had significantly reduced antioxidant levels and significantly increased peroxide levels, which correlated with the fluoride exposure dose. The formation mechanism of oxidative stress, inflammation, and brain damage is mutually causal (38). Due to the disorder of its oxidative and antioxidant capacity levels, the body undergoes a receptor-mediated aseptic inflammatory response and large release of proinflammatory factors, which results in a sustained state of excessive inflammation (39, 40). Here, the body is in the oxidative stress inflammatory microenvironment, and proinflammatory cytokines accelerate the brain injury process (41). We detected the levels of inflammatory factors in rats, and observed a significant increase in the levels of inflammatory factors in the fluoride-exposed groups, which indicated a mutual relationship between oxidative stress, inflammation, and brain damage caused by excessive fluoride intake.

Learning and memory abilities are closely related to the central cholinergic system and are believed to be directly related to learning and memory through interactions with the brain (42). The stability of chemicals in brain tissue affects the thinking, learning, memory, and behavioral changes of normal individuals (43). An important central nervous system neurotransmitter, AchE is a hydrolase of the cholinergic neurotransmitter acetylcholine, and is directly involved in important functions such as neural function regulation, muscle movement, brain thinking, and memory (44). Fluorosis significantly affects α7 nicotinic acetylcholine receptors (n-AChR) and muscarinic acetylcholine receptor (m-AChR) expression in rat brain tissue, thereby affecting learning and memory abilities (45). Our data demonstrated that fluorosis significantly affected AchE and ChAT activity. Furthermore, our results indicated that the changes in learning, memory, and cognitive function of the fluorosis rats were related to AchE and ChAT activity and were influenced by the fluoride exposure dose.

The effect of fluorosis on intelligence may involve multiple different pathways, and the mechanisms of its effects are very complex. After it crosses the blood–brain barrier, fluoride can affect and change the cerebellum, hippocampus, and cerebral cortex to varying degrees, thereby affecting intellectual development (46, 47). The ultrastructural pathological results indicated that fluoride exerted a significant damaging effect on the blood–brain barrier, mainly manifested as astrocyte foot edema, mitochondrial degeneration, and poor function of microvascular endothelial cells. The damaging effect of fluoride on the blood–brain barrier might further increase its accumulation in brain tissue, thereby exacerbating fluoride damage to the brain. Chemical toxin damage to the central nervous system inhibits brain cell proliferation and differentiation, inhibits AchE activity in brain tissue, leads to decreased neurotransmitter synthesis, and thereby affects learning and memory abilities, which was consistent with our results.

SIRT1 is a histone deacetylase mainly expressed in the hippocampal neurons in brain tissue. SIRT1 participates in memory formation, neuroplasticity, and axonal and neuronal protection (48) and regulates inflammatory and oxidative stress responses in the brain, exerting neuroprotective effects (49). SIRT1 enhances BDNF transcriptional activation by regulating BDNF expression, which renders it important in learning, memory, and emotional regulation (50). Animal experiments demonstrated that SIRT1–BDNF signaling pathway activation improved the learning and memory function of vascular cognitive impairment rats (51). BDNF is a neurotrophin family member, a key factor in sympathetic nervous system development, and is known as the fertilizer of the brain (52). BDNF helps the brain generate new neural connections, repair damaged brain cells, and protect healthy brain cells (53). In the brain, BDNF is mainly present in the hippocampus, cerebral cortex and basal forebrain, which are related to learning, memory, recall, and deeper thinking. BDNF binding to its tyrosine kinase receptor TrkB activates PI3K, MAPK, and NF-κB (54, 55). The combination of BDNF and TrkB activates various intracellular signal cascade reactions, then regulates synaptic transmission, promotes cell survival and proliferation, and is neuroprotective (56, 57). This study clarified whether the brain damage and decreased learning and memory abilities caused by fluorosis are related to SIRT1-mediated BDNF–TrkB induced intracellular signaling cascade reactions. The results demonstrated that the fluorosis rat hippocampus had significantly reduced SIRT1 and BDNF–TrkB protein levels, decreased PI3K–Akt and MAPK–NF-κB, and significantly increased apoptotic protein levels, which induced brain damage and affected the rats’ learning and memory abilities.

## 5. Conclusion

Fluoride can accumulate in the brain through the blood–brain barrier, and seriously affect brain memory function. This study provided a better understanding of the pathological mechanisms and learning and memory abilities of fluorosis on brain injury. Excessive fluoride intake in the rats led to peroxide accumulation, causing a receptor-mediated sterile inflammatory response and large release of proinflammatory factors, which directly damaged brain tissue and affected AchE and ChAT activity. Pathological changes in the tissue structure confirmed the progression of brain injury. Protein level validation determined that SIRT1 mediated the BDNF–TrkB signaling pathway to cause a cascade reaction, which led to increased apoptotic protein levels in brain tissue. Further analysis revealed that the SIRT1-mediated decrease in BDNF–TrkB protein levels was involved in the brain damage and learning and memory abilities of the fluorosis rats and negatively correlated with the fluoride exposure dose. Currently, there is no effective treatment method for the tissue damage caused by fluorosis. Therefore, the effective method for preventing and treating fluorosis damage is to control fluoride intake.

## Data availability statement

The datasets presented in this study can be found in online repositories. The names of the repository/repositories and accession number(s) can be found in the article/supplementary material.

## Ethics statement

The animal study was approved by The First Affiliated Hospital of Guizhou University of Traditional Chinese Medicine. The study was conducted in accordance with the local legislation and institutional requirements.

## Author contributions

FW, XZ, DM, DT, YLi, and YLiu conceived of and designed the study, had full access to all data in the study and take responsibility for the integrity of the data and accuracy of data analyses. FW, DT, and YLi wrote the manuscript. ZH, NM, WX, and YLiu revised the manuscript critically. CZ, BY, TT, and MT undertook statistical analyses. All authors contributed to the article and approved the submitted version.

## Funding

This work was supported by the Natural Science Foundation of Guizhou Province [grant numbers QianKeHe Support (2022)181]; the Natural Science Foundation of Guiyang City [grant numbers (2022)4–3-2, (2022)4–3-10, and (2022)4–3-11]; and the Project Foundation of Guizhou Administration of Traditional Chinese Medicine (grant number QZYYXG-2021-5). The funders of the study had no role in study design, data collection, data analysis, data interpretation, or writing of the report.

## Conflict of interest

The authors declare that the research was conducted in the absence of any commercial or financial relationships that could be construed as a potential conflict of interest.

## Publisher’s note

All claims expressed in this article are solely those of the authors and do not necessarily represent those of their affiliated organizations, or those of the publisher, the editors and the reviewers. Any product that may be evaluated in this article, or claim that may be made by its manufacturer, is not guaranteed or endorsed by the publisher.

## References

[1] Wei W Pang S Sun D. The pathogenesis of endemic fluorosis: research progress in the last 5 years. J Cell Mol Med. (2019) 23:2333–42. doi: 10.1111/jcmm.14185, PMID: 30784186 PMC6433665

[2] Amini M Mueller K Abbaspour KC Rosenberg T Afyuni M Møller KN. Statistical modeling of global geogenic fluoride contamination in groundwaters. Environ Sci Technol. (2008) 42:3662–8. doi: 10.1021/es071958y, PMID: 18546705

[3] Liu J Yang S Luo MJ Chen T Ma XJ Tao N. Association between dietary patterns and fluorosis in Guizhou. China Front Nutr. (2020) 6:189. doi: 10.3389/fnut.2019.00189, PMID: 32039225 PMC6985547

[4] Tao N Li L Chen Q Sun Z Yang Q Cao D. Association between antioxidant nutrients, oxidative stress-related gene polymorphism and skeletal fluorosis in Guizhou. China Front Public Health. (2022) 10:849173. doi: 10.3389/fpubh.2022.849173, PMID: 35646794 PMC9140744

[5] Wang F Li Y Tang D Zhao J Yang X Liu Y. Effects of water improvement and defluoridation on fluorosis-endemic areas in China: A meta-analysis. Environ Pollut. (2021) 270:116227. doi: 10.1016/j.envpol.2020.116227, PMID: 33333408

[6] Niu R Chen H Manthari RK Sun Z Wang J Zhang J. Effects of fluoride on synapse morphology and myelin damage in mouse hippocampus. Chemosphere. (2018) 194:628–33. doi: 10.1016/j.chemosphere.2017.12.027, PMID: 29241138

[7] Liu H Gao Y Sun L Li M Li B Sun D. Assessment of relationship on excess fluoride intake from drinking water and carotid atherosclerosis development in adults in fluoride endemic areas. China Int J Hyg Environ Health. (2014) 217:413–20. doi: 10.1016/j.ijheh.2013.08.001, PMID: 24012047

[8] Srivastava S Flora SJS. Fluoride in drinking water and skeletal fluorosis: a review of the global impact. Curr Environ Health Rep. (2020) 7:140–6. doi: 10.1007/s40572-020-00270-9, PMID: 32207100

[9] Wang Q Cui KP Xu YY Gao YL Zhao J Li DS. Coal-burning endemic fluorosis is associated with reduced activity in antioxidative enzymes and cu/Zn-SOD gene expression. Environ Geochem Health. (2014) 36:107–15. doi: 10.1007/s10653-013-9522-2, PMID: 23567976

[10] Suzuki M Bandoski C Bartlett JD. Fluoride induces oxidative damage and SIRT1/autophagy through ROS-mediated JNK signaling. Free Radic Biol Med. (2015) 89:369–78. doi: 10.1016/j.freeradbiomed.2015.08.015 26431905 PMC4684823

[11] Babu S Manoharan S Ottappilakkil H Perumal E. Role of oxidative stress-mediated cell death and signaling pathways in experimental fluorosis. Chem Biol Interact. (2022) 365:110106. doi: 10.1016/j.cbi.2022.110106 35985521

[12] Maheshwari N Qasim N Anjum R Mahmood R. Fluoride enhances generation of reactive oxygen and nitrogen species, oxidizes hemoglobin, lowers antioxidant power and inhibits transmembrane electron transport in isolated human red blood cells. Ecotoxicol Environ Saf. (2021) 208:111611. doi: 10.1016/j.ecoenv.2020.111611 33396131

[13] Lee YS Kim JW Osborne O Oh DY Sasik R Schenk S. Increased adipocyte O2 consumption triggers HIF-1α, causing inflammation and insulin resistance in obesity. Cells. (2014) 157:1339–52. doi: 10.1016/j.cell.2014.05.012 PMC4114226 24906151

[14] Tu C Lu H Zhou T Zhang W Deng L Cao W. Promoting the healing of infected diabetic wound by an anti-bacterial and nano-enzyme-containing hydrogel with inflammation-suppressing, ROS-scavenging, oxygen and nitric oxide-generating properties. Biomaterials. (2022) 286:121597. doi: 10.1016/j.biomaterials.2022.121597, PMID: 35688112

[15] Sudheer SP Sen U Kapoor S Ranade AV Chowdhury CR Bose B. Sodium fluoride induced skeletal muscle changes: degradation of proteins and signaling mechanism. Environ Pollut. (2019) 244:534–48. doi: 10.1016/j.envpol.2018.10.034, PMID: 30384060

[16] Ren C Li HH Zhang CY Song XC. Effects of chronic fluorosis on the brain. Ecotoxicol Environ Saf. (2022) 244:114021. doi: 10.1016/j.ecoenv.2022.114021, PMID: 36049331

[17] Agalakova NI Nadei OV. Inorganic fluoride and functions of brain. Crit Rev Toxicol. (2020) 50:28–46. doi: 10.1080/10408444.2020.1722061, PMID: 32073339

[18] Saeed M Malik RN Kamal A. Fluorosis and cognitive development among children (6-14 years of age) in the endemic areas of the world: a review and critical analysis. Environ Sci Pollut Res Int. (2020) 27:2566–79. doi: 10.1007/s11356-019-06938-6, PMID: 31867690

[19] David C. Bellinger. Is fluoride potentially neurotoxic. JAMA Pediatr. (2019) 173:915–7. doi: 10.1001/jamapediatrics.2019.1728, PMID: 31424483

[20] Wang J Yue B Zhang X Guo X Sun Z Niu R. Effect of exercise on microglial activation and transcriptome of hippocampus in fluorosis mice. Sci Total Environ. (2021) 760:143376. doi: 10.1016/j.scitotenv.2020.143376, PMID: 33172640

[21] El Hayek L Khalifeh M Zibara V Abi Assaad R Emmanuel N Karnib N. Lactate mediates the effects of exercise on learning and memory through SIRT1-dependent activation of hippocampal brain-derived neurotrophic factor (BDNF). J Neurosci. (2019) 39:2369–82. doi: 10.1523/JNEUROSCI.1661-18.2019, PMID: 30692222 PMC6435829

[22] Mirshafa A Mohammadi H Shokrzadeh M Mohammadi E Talebpour Amiri F Shaki F. Tropisetron protects against brain aging via attenuating oxidative stress, apoptosis and inflammation: the role of SIRT1 signaling. Life Sci. (2020) 248:117452. doi: 10.1016/j.lfs.2020.117452, PMID: 32088214

[23] Marosi K Mattson MP. BDNF mediates adaptive brain and body responses to energetic challenges. Trends Endocrinol Metab. (2014) 25:89–98. doi: 10.1016/j.tem.2013.10.006, PMID: 24361004 PMC3915771

[24] Liu B Zhang Y Yang Z Liu M Zhang C Zhao Y. ω-3 DPA protected neurons from Neuroinflammation by balancing microglia M1/M2 polarizations through inhibiting NF-κB/MAPK p38 signaling and activating neuron-BDNF-PI3K/AKT pathways. Mar Drugs. (2021) 19:587. doi: 10.3390/md19110587, PMID: 34822458 PMC8619469

[25] Tung WH Lee IT Hsieh HL Yang CM. EV71 induces COX-2 expression via c-Src/PDGFR/PI3K/Akt/p42/p44 MAPK/AP-1 and NF-kappaB in rat brain astrocytes. J Cell Physiol. (2010) 224:376–86. doi: 10.1002/jcp.22133, PMID: 20333648

[26] James P Harding M Beecher T Browne D Cronin M Guiney H. Impact of reducing water fluoride on dental caries and fluorosis. J Dent Res. (2021) 100:507–14. doi: 10.1177/0022034520978777, PMID: 33345672

[27] Qiao L Liu X He Y Zhang J Huang H Bian W. Progress of Signaling Pathways, Stress Pathways and Epigenetics in the Pathogenesis of Skeletal Fluorosis. Int J Mol Sci. (2021) 22:11932. doi: 10.3390/ijms222111932, PMID: 34769367 PMC8584317

[28] Lavalle-Carrasco J Vergara-Onofre M González-González R Bologna-Molina R Isiordia-Espinoza MA Gaona E. A systematic review and Meta-analysis of the relationship between the severity of dental fluorosis and fluoride biomarkers in endemic areas. Biol Trace Elem Res. (2023) 201:1051–62. doi: 10.1007/s12011-022-03227-1, PMID: 35397104

[29] Davis S Butcher SP Morris RG. The NMDA receptor antagonist D-2-amino-5-phosphonopentanoate (D-AP5) impairs spatial learning and LTP in vivo at intracerebral concentrations comparable to those that block LTP in vitro. J Neurosci. (1992) 12:21–34. doi: 10.1523/JNEUROSCI.12-01-00021.1992, PMID: 1345945 PMC6575679

[30] Lissner LJ Wartchow KM Toniazzo AP Gonçalves CA Rodrigues L. Object recognition and Morris water maze to detect cognitive impairment from mild hippocampal damage in rats: a reflection based on the literature and experience. Pharmacol Biochem Behav. (2021) 210:173273. doi: 10.1016/j.pbb.2021.173273, PMID: 34536480

[31] Ding Y Gao Y Sun H Han H Wang W Ji X. The relationships between low levels of urine fluoride on children's intelligence, dental fluorosis in endemic fluorosis areas in Hulunbuir, Inner Mongolia, China. J Hazard Mater. (2011) 186:1942–6. doi: 10.1016/j.jhazmat.2010.12.097, PMID: 21237562

[32] Wang Y Branicky R Noë A Hekimi S. Superoxide dismutases: dual roles in controlling ROS damage and regulating ROS signaling. J Cell Biol. (2018) 217:1915–28. doi: 10.1083/jcb.201708007, PMID: 29669742 PMC5987716

[33] Zhong N Yao Y Ma Y Meng X Sowanou A Pei J. Effects of fluoride on oxidative stress markers of lipid, gene, and protein in rats. Biol Trace Elem Res. (2021) 199:2238–46. doi: 10.1007/s12011-020-02336-z, PMID: 32789643

[34] Chan JYH Chan SHH. Differential impacts of brain stem oxidative stress and nitrosative stress on sympathetic vasomotor tone. Pharmacol Ther. (2019) 201:120–36. doi: 10.1016/j.pharmthera.2019.05.015, PMID: 31153955

[35] Trist BG Hilton JB Hare DJ Crouch PJ Double KL. Superoxide dismutase 1 in health and disease: how a frontline antioxidant becomes neurotoxic. Angew Chem Int Ed Engl. (2021) 60:9215–46. doi: 10.1002/anie.202000451 32144830 PMC8247289

[36] Ottappilakkil H Babu S Balasubramanian S Manoharan S Perumal E. Fluoride induced neurobehavioral impairments in experimental animals: a brief review. Biol Trace Elem Res. (2023) 201:1214–36. doi: 10.1007/s12011-022-03242-2 35488996

[37] Souza-Monteiro D Ferreira MKM Bittencourt LO Aragão WAB Oliveira IG Maia CSF. Intrauterine and postnatal exposure to high levels of fluoride is associated with motor impairments, oxidative stress, and morphological damage in the cerebellum of offspring rats. Int J Mol Sci. (2022) 23:8556. doi: 10.3390/ijms23158556, PMID: 35955690 PMC9369436

[38] Chamorro Á Dirnagl U Urra X Planas AM. Neuroprotection in acute stroke: targeting excitotoxicity, oxidative and nitrosative stress, and inflammation. Lancet Neurol. (2016) 15:869–81. doi: 10.1016/S1474-4422(16)00114-9 27180033

[39] Hybertson BM Gao B Bose SK McCord JM. Oxidative stress in health and disease: the therapeutic potential of Nrf2 activation. Mol Asp Med. (2011) 32:234–46. doi: 10.1016/j.mam.2011.10.006, PMID: 22020111

[40] Matyas C Haskó G Liaudet L Trojnar E Pacher P. Interplay of cardiovascular mediators, oxidative stress and inflammation in liver disease and its complications. Nat Rev Cardiol. (2021) 18:117–35. doi: 10.1038/s41569-020-0433-5, PMID: 32999450

[41] Hagberg H Mallard C Ferriero DM Vannucci SJ Levison SW Vexler ZS. The role of inflammation in perinatal brain injury. Nat Rev Neurol. (2015) 11:192–208. doi: 10.1038/nrneurol.2015.13, PMID: 25686754 PMC4664161

[42] Bohnen NI Yarnall AJ Weil RS Moro E Moehle MS Borghammer P. Cholinergic system changes in Parkinson's disease: emerging therapeutic approaches. Lancet Neurol. (2022) 21:381–92. doi: 10.1016/S1474-4422(21)00377-X, PMID: 35131038 PMC8985079

[43] Lee Y Lee TW. Organic synapses for neuromorphic electronics: from brain-inspired computing to sensorimotor Nervetronics. Acc Chem Res. (2019) 52:964–74. doi: 10.1021/acs.accounts.8b00553, PMID: 30896916

[44] Llorca-Torralba M Suárez-Pereira I Bravo L Camarena-Delgado C Garcia-Partida JA Mico JA. Chemogenetic silencing of the locus Coeruleus-basolateral amygdala pathway abolishes pain-induced anxiety and enhanced aversive learning in rats. Biol Psychiatry. (2019) 85:1021–35. doi: 10.1016/j.biopsych.2019.02.018, PMID: 30987747

[45] Dong YT Wang Y Wei N Zhang QF Guan ZZ. Deficit in learning and memory of rats with chronic fluorosis correlates with the decreased expressions of M1 and M3 muscarinic acetylcholine receptors. Arch Toxicol. (2015) 89:1981–91. doi: 10.1007/s00204-014-1408-2 25417050

[46] Qing-Feng S Ying-Peng X Tian-Tong X. Matrix metalloproteinase-9 and p53 involved in chronic fluorosis induced blood-brain barrier damage and neurocyte changes. Arch Med Sci. (2019) 15:457–66. doi: 10.5114/aoms.2019.83294 30899299 PMC6425220

[47] Wang S Zhao Q Li G Wang M Liu H Yu X. The cholinergic system, intelligence, and dental fluorosis in school-aged children with low-to-moderate fluoride exposure. Ecotoxicol Environ Saf. (2021) 228:112959. doi: 10.1016/j.ecoenv.2021.112959, PMID: 34808511

[48] Paraíso AF Mendes KL Santos SH. Brain activation of SIRT1: role in neuropathology. Mol Neurobiol. (2013) 48:681–9. doi: 10.1007/s12035-013-8459-x 23615921

[49] Cong L Lei MY Liu ZQ Liu ZF Ma Z Liu K. Resveratrol attenuates manganese-induced oxidative stress and neuroinflammation through SIRT1 signaling in mice. Food Chem Toxicol. (2021) 153:112283. doi: 10.1016/j.fct.2021.112283, PMID: 34029668

[50] Gao J Wang WY Mao YW Gräff J Guan JS Pan L. A novel pathway regulates memory and plasticity via SIRT1 and miR-134. Nature. (2010) 466:1105–9. doi: 10.1038/nature09271, PMID: 20622856 PMC2928875

[51] Stein S Winnik S Matter CM. Brain-derived neurotrophic factor Val66Met polymorphism in depression and thrombosis: SIRT1 as a possible mediator. Eur Heart J. (2017) 38:1436–8. doi: 10.1093/eurheartj/ehv692 26715164

[52] Treble-Barna A Heinsberg LW Stec Z Breazeale S Davis TS Kesbhat AA. Brain-derived neurotrophic factor (BDNF) epigenomic modifications and brain-related phenotypes in humans: a systematic review. Neurosci Biobehav Rev. (2023) 147:105078. doi: 10.1016/j.neubiorev.2023.105078 36764636 PMC10164361

[53] Cotman CW Berchtold NC. Exercise: a behavioral intervention to enhance brain health and plasticity. Trends Neurosci. (2002) 25:295–301. doi: 10.1016/s0166-2236(02)02143-4 12086747

[54] Li C Sui C Wang W Yan J Deng N Du X. Baicalin attenuates oxygen-glucose deprivation/Reoxygenation-induced injury by modulating the BDNF-TrkB/PI3K/Akt and MAPK/Erk1/2 signaling axes in neuron-astrocyte Cocultures. Front Pharmacol. (2021) 12:599543. doi: 10.3389/fphar.2021.599543 34234667 PMC8255628

[55] Rabie MA Abd El Fattah MA Nassar NN El-Abhar HS Abdallah DM. Angiotensin 1-7 ameliorates 6-hydroxydopamine lesions in hemiparkinsonian rats through activation of MAS receptor/PI3K/Akt/BDNF pathway and inhibition of angiotensin II type-1 receptor/NF-κB axis. Biochem Pharmacol. (2018) 151:126–34. doi: 10.1016/j.bcp.2018.01.047 29428223

[56] Casarotto PC Girych M Fred SM Kovaleva V Moliner R Enkavi G. Antidepressant drugs act by directly binding to TRKB neurotrophin receptors. Cells. (2021) 184:1299–1313.e19. doi: 10.1016/j.cell.2021.01.034 PMC7938888 33606976

[57] Li W Ali T Zheng C He K Liu Z Shah FA. Anti-depressive-like behaviors of APN KO mice involve Trkb/BDNF signaling related neuroinflammatory changes. Mol Psychiatry. (2022) 27:1047–58. doi: 10.1038/s41380-021-01327-3, PMID: 34642455

